# Genome-wide characterization of genetic diversity and population structure of the Chilean Mapuche fowl using DArTseq SNP markers

**DOI:** 10.1016/j.psj.2026.107715

**Published:** 2026-08-27

**Authors:** Mario Díaz-Matus de la Parra, Karla Inostroza, José Antonio Alcalde, Daniela Bustos-Korts, Giovanni Larama, Silvana Bravo

**Affiliations:** a Graduate School, Faculty of Agricultural and Food Sciences, Universidad Austral de Chile, Valdivia, Chile; b Núcleo de Investigación en Producción Alimentaria, Facultad de Recursos Naturales, Universidad Católica de Temuco, Temuco, Chile; c Department of Fruit Cultivation and Enology, Faculty of Agronomy and Natural Systems, Pontificia Universidad Católica de Chile, Santiago 7820436, Chile; d Institute of Plant Production and Plant Health, Faculty of Agricultural and Food Sciences, Universidad Austral de Chile, Valdivia, Chile; e Biocontrol Research Laboratory, Universidad de La Frontera, Temuco 4811230, Chile; f Institute of Animal Production, Faculty of Agricultural and Food Sciences, Universidad Austral de Chile, P.O. Box 567, Valdivia, Chile; g Center for Research on Volcanic Soils, Faculty of Agricultural and Food Sciences, Universidad Austral de Chile, Valdivia, Chile

**Keywords:** Mapuche fowl, Genetic diversity, Population structure, Poultry genetics

## Abstract

The Mapuche fowl is one of the most representative animal genetic resources in Chile, traditionally maintained in small-scale backyard production systems. Despite its cultural and productive relevance, genomic studies evaluating its genetic diversity and population structure remain limited. The objective of this study was to characterize the genetic diversity and population structure of the Mapuche fowl using genome-wide SNP markers obtained through DArTseq technology.

A total of 91 chickens representing seven populations—including three Mapuche fowl subtypes, Gallina de la Pasión, two commercial breeds, and one commercial hybrid line, were analyzed using 43,017 high-quality SNP markers generated through DArTseq. Genetic diversity parameters, population differentiation (FST), multivariate analyses (PCA and DAPC), phylogenetic relationships, population structure based on sNMF, and analysis of molecular variance (AMOVA) were evaluated.

The results revealed intermediate levels of genetic diversity across populations, with expected heterozygosity ranging from 0.166 to 0.236. The Mapuche fowl varieties showed the highest levels of genetic diversity and formed a closely related genetic group with low levels of differentiation (FST = 0.026–0.032). In contrast, commercial breeds exhibited greater genetic divergence. Population structure analyses consistently showed clear differentiation between commercial and local populations, while the Mapuche fowl varieties shared ancestry components and clustered closely together. AMOVA indicated that most genetic variation occurred within populations (72.93%), whereas 13.25% was attributed to differences among populations.

These findings highlight the genetic uniqueness of the Mapuche fowl and provide a genomic basis for its conservation and sustainable management as a valuable poultry genetic resource in Chile.

## Introduction

The characterization of animal genetic resources (Fig. 1) constitutes a fundamental tool for their proper management, conservation, and sustainable use. This characterization is generally structured into two complementary approaches: phenotypic characterization and genetic characterization (Delgado et al., 2001; Rogberg et al., 2015; Brito et al., 2021; González Ariza et al., 2021; Zhi et al., 2025). Genetic characterization of animal genetic resources allows the quantification of genetic variability within and among populations, the evaluation of population structure, the detection of genetic differentiation processes, and the estimation of phylogenetic relationships among populations or breeds (Caballero and Rodriguez-Ramilo, 2010; Fernández and Bennewitz, 2017; López-Cortegano et al., 2019; Kierkegaard et al., 2020; Díaz-Matus de la Parra et al., 2024). In this context, the Mapuche fowl is one of the most representative animal genetic resources in Chile. This population originated in the historical territory of the Mapuche people and has been maintained for generations within traditional small-scale production systems, which has contributed to the conservation of a high level of phenotypic and genetic diversity (Moya and Montero, 2007; Moya et al., 2009; Alcalde, 2016; Díaz-Matus de la Parra et al., 2024). Within this population, three main subtypes are recognized: Kollonka, characterized by the absence of a tail (rumpless) (Castello, 1924; Dunn and Landauer, 1934; Noorai et al., 2012; Wragg et al., 2012, 2013; Wang et al., 2013; Freese et al., 2014; Alcalde, 2016; Díaz-Matus de la Parra et al., 2024); Ketro, which exhibits ear tufts or lateral feather appendages (Bustos, 1922; Somes, 1978; Pabilonia and Somes, 1983; Noorai et al., 2012; Alcalde, 2016; Díaz-Matus de la Parra et al., 2024) and Kollonka de Aretes, which results from the crossing of the two former subtypes, the rumpless Kollonka and the ear-tufted Ketro. This latter population gave rise to the standard of the Araucana breed recognized in North America and Europe (Castello, 1924; Wilhelm, 1963; Alcalde, 2016; Díaz-Matus de la Parra et al., 2024). The most distinctive trait of the Mapuche fowl is the blue-green eggshell coloration. This trait has attracted considerable productive and commercial interest, particularly in markets oriented toward differentiated eggs, adding economic value to this population beyond its cultural and genetic significance (Castello, 1924; Punnett, 1933; Zhao Rui et al., 2007; González Ariza et al., 2019; Altmann et al., 2023; Chen et al., 2023). Despite its cultural, historical, and productive relevance, the Mapuche fowl is mainly maintained in backyard production systems, where breeding practices are often poorly controlled (Moya and Montero, 2007; Moya et al., 2009; Alcalde, 2016; Díaz-Matus de la Parra et al., 2024). In these systems, the exchange of birds among producers is common, as well as the occasional introduction of commercial breeds or productive hybrids. These practices may lead to crossbreeding processes, uncontrolled gene flow, and potential genetic erosion of local populations (Díaz-Matus de la Parra et al., 2024). Consequently, the genetic characterization of these populations is essential to understand their genetic structure, estimate their levels of genetic diversity, admixture, and inbreeding, and to generate information that supports the design of conservation strategies and the sustainable management of this animal genetic resource.

**Fig. 1:**
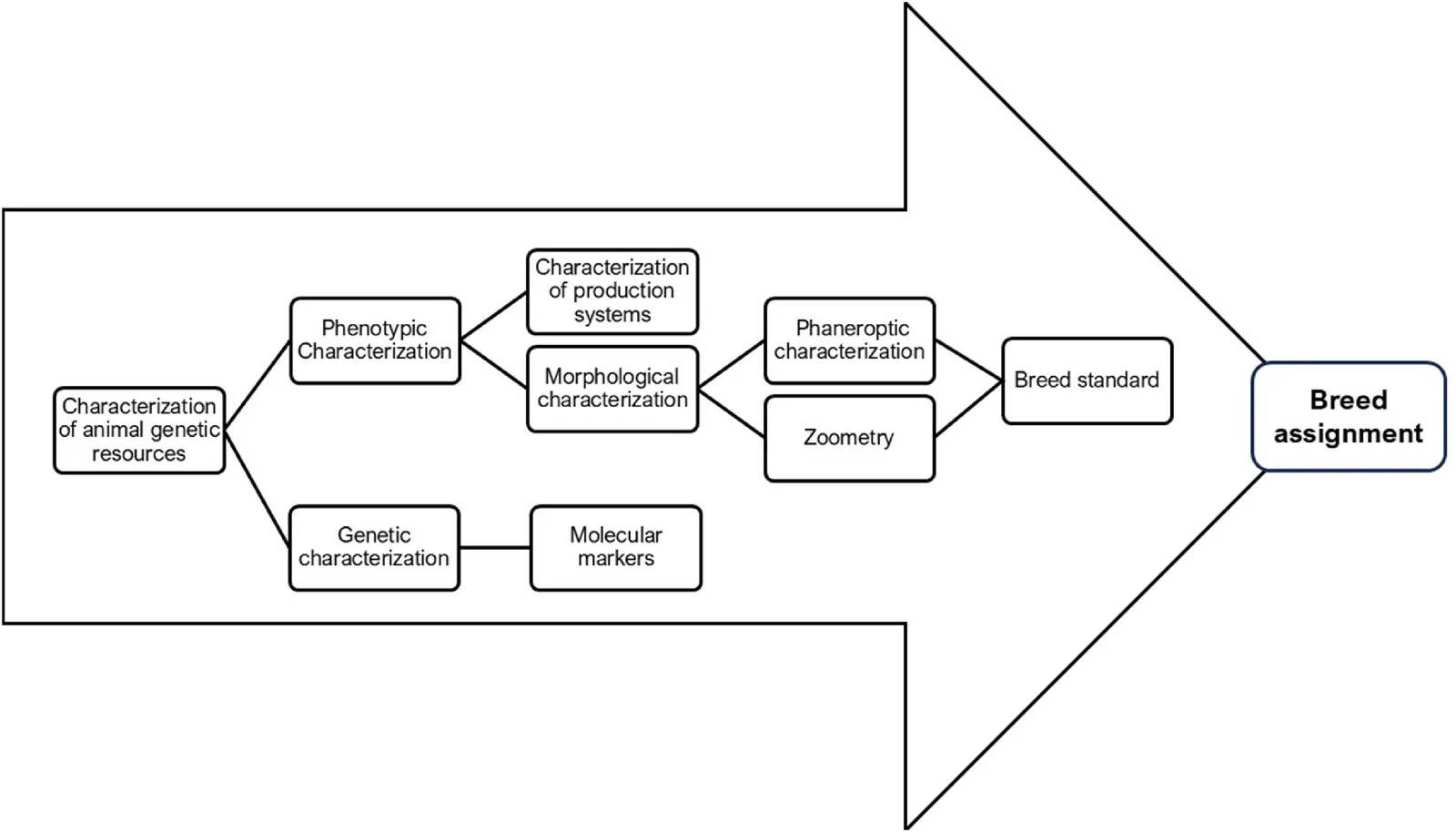
Characterization process of an animal genetic resource.

In addition to the Mapuche fowl subtypes, Chilean backyard systems maintain other historically recognized chicken populations, including Gallina de la Pasión, locally known as “Kike” or “Kikiriki.” This dwarf chicken has a recognizable phenotypic identity and is primarily maintained for ornamental purposes. Mitochondrial evidence has associated Chilean chickens with maternal lineages of Asian and European origin and has revealed haplotype sharing among local populations, suggesting a complex history of common ancestry and introgression (Gongora et al., 2008; Díaz-Matus de la Parra et al., 2024). However, the genome-wide diversity and genetic relationships of Gallina de la Pasión have not previously been evaluated.

Recent advances in high-throughput genotyping have substantially increased the resolution available for assessing genetic diversity in domestic and wild populations. Single nucleotide polymorphisms (SNPs) are particularly informative because they are abundant and widely distributed across the genome, stable, and suitable for detecting genetic variation within and among populations (Hillel et al., 2007; Groeneveld et al., 2010; FAO, 2011; Tan et al., 2024). Consequently, genome-wide SNP datasets are increasingly used to investigate population structure, genetic relationships, demographic history, and animal genetic resources relevant to breeding and conservation programs (Groeneveld et al., 2010; FAO, 2011; Tan et al., 2024).

Diversity Arrays Technology (DArT) was initially developed as a hybridization-based genotyping method capable of detecting numerous polymorphic loci throughout the genome without requiring prior sequence information (Jaccoud et al., 2001; Wenzl et al., 2004). Its integration with next-generation sequencing led to DArTseq, a reduced-representation sequencing approach that combines genome complexity reduction, typically through restriction-enzyme digestion, with high-throughput sequencing. This strategy enables the simultaneous discovery and genotyping of codominant SNPs and dominant SilicoDArT markers distributed across the genome (Ferguson et al., 2012; Kilian et al., 2012; Alam et al., 2018). The capacity to generate large numbers of informative markers, together with its high throughput, reproducibility, and cost-effectiveness, has supported the use of DArTseq in genetic diversity assessment, population structure analysis, genetic mapping, and germplasm characterization across a broad range of species, including those with limited prior genomic information (Ferguson et al., 2012; Alam et al., 2018; Pierre et al., 2022). More recently, its application to domestic chicken populations has demonstrated its utility for assessing genome-wide genetic variability and identifying patterns of differentiation and admixture, particularly among locally managed populations (Mujyambere et al., 2024, 2025).

Despite the cultural, productive, and genetic importance of Mapuche fowl, its diversity and population structure have not previously been characterized using genome-wide SNP markers. Previous microsatellite analyses identified close genetic relationships and admixture among its principal subtypes but provided limited resolution for evaluating genome-wide differentiation (Díaz-Matus de la Parra et al., 2024). Moreover, no genome-wide characterization is currently available for Gallina de la Pasión. DArTseq-derived SNPs therefore provide an appropriate framework for resolving the genetic relationships among these local populations and comparing them with commercial genetic groups. Accordingly, this study aimed to characterize the genetic diversity and population structure of three Mapuche fowl subtypes and Gallina de la Pasión using genome-wide DArTseq SNP markers, with Brahma, Barred Plymouth Rock, and ISA Brown included as comparative populations.

## Materials and methods

### Ethics statement

All procedures involving animals were conducted in accordance with international guidelines for the ethical use and care of animals in research and complied with institutional regulations on animal welfare. The study protocol was reviewed and approved by the Institutional Committee for the Care and Use of Animals (CICUA) of the Universidad Austral de Chile, under animal management protocol No. 570/2025. All efforts were made to minimize stress and discomfort the animals during handling and sampling procedures.

### Study area

The study area extended from the Coquimbo Region to the Aysén Region in Chile, covering a wide latitudinal range in the country, as shown in Fig. 2, from La Serena (29°54′0″ South, 71°15′0″ West, Coquimbo region) to Coyhaique (Aysén Region, southern Chilean Patagonia 45°34′0″ South, 72°04′0″ West).

**Fig. 2:**
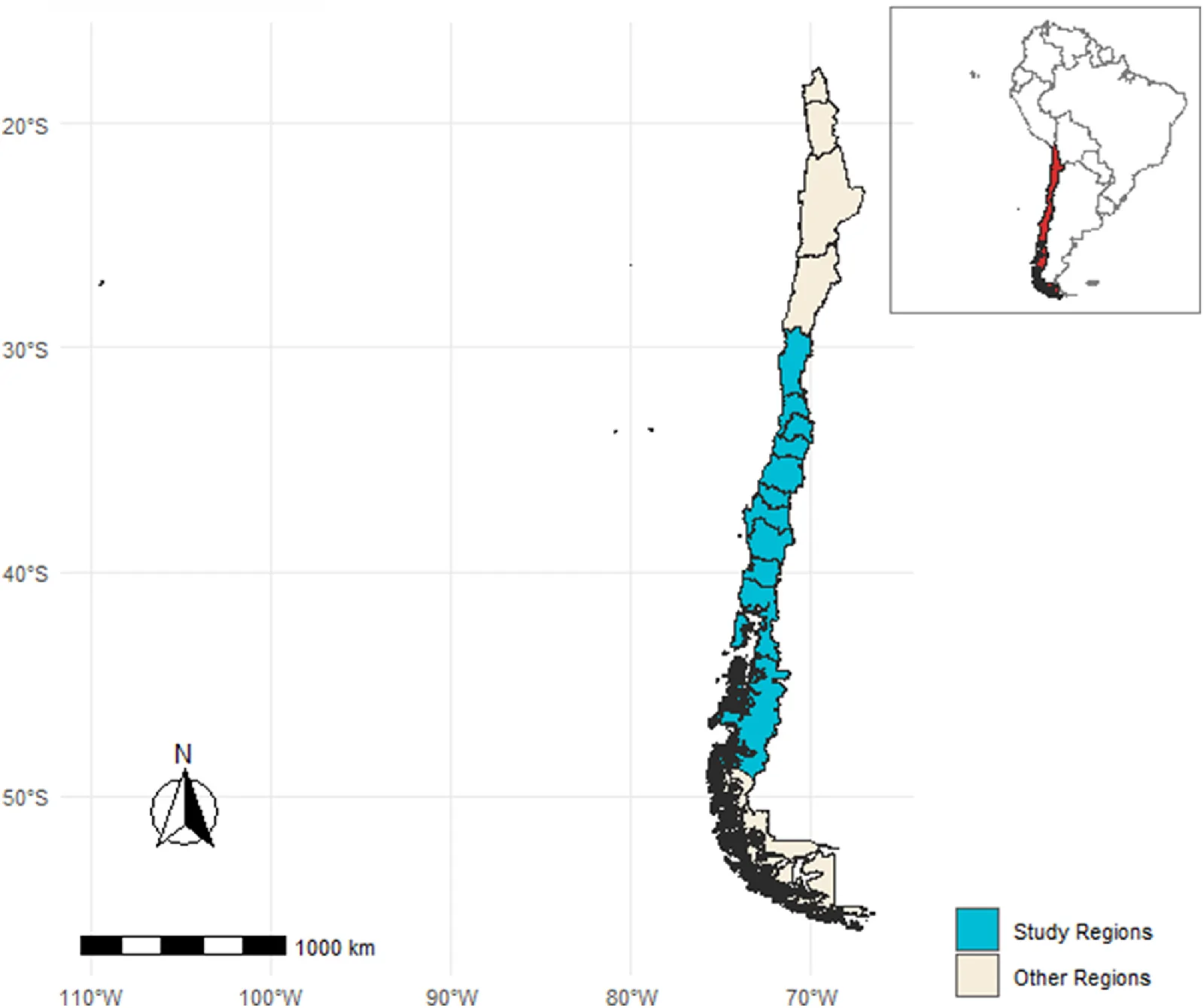
Map of Chile showing the regions included in the study. The regions highlighted in cyan correspond to: Coquimbo Region, Valparaíso Region, Metropolitan Region, Libertador General Bernardo O’Higgins Region, Maule Region, Ñuble Region, Biobío Region, La Araucanía Region, Los Ríos Region, Los Lagos Region, and Aysén del General Carlos Ibáñez del Campo Region.

### Animals and sample collection

A total of 91 chickens belonging to seven different breeds: Kollonka, Ketro, Kollonka de Aretes, Gallina de la Pasión, Brahma, Barred Plymouth Rock and ISA Brown line were genotyped. Birds of both sexes were sampled, as detailed in Table 1. For six of the studied breeds, biological material for DNA extraction was obtained from peripheral blood collected from the brachial vein. In contrast, in the Gallina de la Pasión breed, freshly plucked feathers with intact follicles were used as the DNA source due to the smaller body size and lower body weight of these birds, to minimize handling stress and reduce the invasiveness of sampling. Feathers were collected from the pectoral region, wings, and/or tail, immediately placed in hermetically sealed plastic bags (Ziploc® type), and individually labeled with the following information: breed, animal identification number, sex, producer name, and farm location. All feather samples were subsequently stored at −20 °C until further processing. For the remaining breeds, peripheral blood was collected via puncture of the brachial vein, using sterile syringes and 27-gauge hypodermic needles, with Vacutainer® tubes containing EDTA as anticoagulant. The volume collected was equivalent to 0.1% of the bird’s live weight, in accordance with bioethical guidelines for blood sampling in domestic poultry. Each blood sample was labeled with the same identifying information described above and stored at −20 °C until molecular analysis.

**Table 1:** Breed, sex and number of individuals among the studied populations.

Breed	Sex	Number of individuals
Kollonka	Male	12
	Female	3
Ketro	Male	8
	Female	6
Kollonka de Aretes	Male	13
	Female	2
Gallina de la Pasión	Male	6
	Female	7
Brahma	Male	4
	Female	9
Barred Plymouth Rock	Male	4
	Female	9
ISA Brown	Female	8
	**TOTAL**	**91**

### Collection of samples and DNA extraction

Biological samples consisted of peripheral blood collected using the Vacutainer® system and freshly plucked feathers with intact follicles, which were stored at −20°C until analysis. DNA extraction was carried out with the ZYMO RESEARCH Quick-DNA™ Miniprep Plus Kit. DNA presence and quality were verified by 1% agarose gel electrophoresis. In addition, DNA concentration and purity were assessed using a NanoQuant Infinite® 200 PRO spectrophotometer (Tecan Trading AG, Switzerland). Samples with an absorbance ratio (260/280) greater than 1.8 were considered suitable for amplification. Stock DNA was then diluted to a final concentration of 10 ng/μL, and the final dilutions were stored at −20°C for future use.

### DArTseq genotyping and SNP identification

For genomic analysis, the DArTseq™ (Diversity Arrays Technology sequencing) platform was used (https://www.diversityarrays.com/). Library construction was performed through genomic complexity reduction, which involved the digestion of genomic DNA with specific restriction enzymes followed by the ligation of barcoded adapters to the resulting fragments. Subsequently, the adapter-ligated fragments were amplified by PCR to generate genomic libraries. The constructed libraries were sequenced using single-end reads of 77 base pairs on the Illumina HiSeq 2500 next-generation sequencing platform. This sequencing approach is based on the genotyping-by-sequencing (GBS) methodology implemented in DArTseq technology, which enables the rapid and cost-effective generation of high-quality genomic profiles across thousands of loci, even in complex genomes (Kilian et al., 2012). Identification and scoring of DArTseq markers were performed using DArTsoft14, a proprietary marker-calling pipeline based on classification algorithms developed by Diversity Arrays Technology Pty Ltd. This pipeline processes raw sequencing reads to identify polymorphic loci and perform genotype calling while calculating marker quality metrics such as call rate, reproducibility, and allele frequencies. SNP genotypes generated through DArTseq were coded numerically according to allele dosage, where 0 represents the homozygous reference genotype, 1 the heterozygous genotype, and 2 the homozygous alternative genotype, while missing data were coded as NA. Finally, the identified SNP markers were aligned to the chicken reference genome (*Gallus gallus* genome assembly) to determine their chromosomal positions and genomic locations.

### SNP quality control and filtering

Genotype data obtained from the DArTseq platform were subjected to quality control filtering prior to population genetic analyses. SNP data were imported into the R statistical environment as a genlight object using functions implemented in the dartR package (Gruber et al., 2018), which allows efficient handling and analysis of large SNP datasets. Quality filtering was applied to remove markers with low reliability. First, locus call rate was evaluated, defined as the proportion of individuals successfully genotyped at each locus. Markers with a call rate lower than 0.90 were removed to reduce the influence of missing data on downstream analyses. A second filtering step was applied based on minor allele frequency (MAF), and loci with MAF lower than 0.05 were excluded to eliminate markers with extremely low allele frequencies that contribute limited information for population genetic analyses. In addition, individual call rate was evaluated to assess sample-level genotyping completeness; however, individual call rate was not used as an exclusion criterion, and all sampled individuals were retained in the dataset. After applying all filtering criteria, the final dataset consisted of 43,017 high-quality SNP markers and 91 individuals, which were retained for subsequent analyses of genetic diversity and population structure. All quality control and filtering procedures were performed in R using the dartR package, ensuring a reproducible workflow for SNP data processing.

### Genetic diversity analysis

Genetic diversity within populations was estimated using standard population genetic parameters derived from the filtered SNP dataset. The SNP data were imported into the R statistical environment as a genlight object and converted into a genotype matrix coded as 0, 1, and 2 according to allele dosage, where 0 represents the homozygous reference genotype, 1 the heterozygous genotype, and 2 the homozygous alternative genotype, while missing data were coded as NA. For each population, observed heterozygosity (Ho), expected heterozygosity (He), and the inbreeding coefficient (FIS) were calculated from allele frequencies across loci. Specifically, allele frequencies were estimated from the mean genotype dosage per locus, Ho was calculated as the proportion of heterozygous genotypes, He was estimated as 2p(1−p), and FIS was derived as 1−(Ho/He). These parameters allow the assessment of within-population genetic variation and deviations from Hardy–Weinberg expectations (Nei, 1978; Hartl and Clark, 1989; Wills, 2007). In addition, the distribution of alternate SNP allele counts was evaluated within each population by summing allele dosages across individuals at each locus, to describe patterns of allele frequency variation among breeds and assess the overall genomic variability present in the dataset. All analyses were conducted in the R statistical environment using functions implemented in the dartR (Gruber et al., 2018) and adegenet (Jombart, 2008) packages for SNP data handling and processing, together with custom calculations applied to the filtered genotype matrix.

### Population differentiation

Genetic differentiation among populations was assessed using fixation index (FST) values, which quantify the degree of genetic divergence between pairs of populations based on allele frequency differences (Wright, 1951; Weir and Cockerham, 1984). FST values provide a measure of population structure and allow identification of genetically differentiated groups. In addition, Nei’s genetic distances (Nei, 1972) were calculated to evaluate the genetic relationships among the analyzed populations. These distances were used to construct genetic distance matrices that served as the basis for subsequent graphical representations. The resulting differentiation matrices were visualized through heatmaps of pairwise FST values and were also used to infer phylogenetic relationships among populations based on genetic distances.

### Population structure and admixture analysis

Population structure and admixture patterns were additionally evaluated using sparse nonnegative matrix factorization (sNMF), as implemented in the R package LEA (Frichot et al., 2014; Frichot and François, 2015; Gain and François, 2021) or this purpose, the filtered SNP dataset, comprising 91 individuals and 43,017 SNPs, was converted to the geno format and analyzed using the sNMF function. The analysis was performed considering values of K from 1 to 10, with 100 independent repetitions for each K, to ensure consistency and stability of the inferred ancestry coefficients. The most likely number of ancestral populations was inferred by comparing cross-entropy values across K values and selecting the solution with the lowest cross-entropy. Individual ancestry coefficients (Q matrix) were then extracted from the best run for each K. To facilitate interpretation of ancestry patterns, individuals were ordered according to their population of origin in the graphical outputs.

### Multivariate analyses

The genetic structure of the analyzed populations was explored using multivariate statistical approaches. A Principal Component Analysis (PCA) was performed to reduce the dimensionality of the SNP dataset and to visualize patterns of genetic variation among individuals without assuming prior population structure (Jolliffe and Cadima, 2016; Kherif and Latypova, 2020). Additionally, a Discriminant Analysis of Principal Components (DAPC) was performed to maximize genetic separation among predefined groups while minimizing variation within groups (Jombart et al., 2010). This method combines PCA with discriminant analysis to identify genetic clusters and evaluate the assignment of individuals to their respective populations. Both PCA and DAPC analyses were conducted in the R environment using the adegenet package (Jombart, 2008).

### Analysis of molecular variance (AMOVA)

The partitioning of genetic variation within and among populations was evaluated using an Analysis of Molecular Variance (AMOVA) based on the filtered SNP dataset. AMOVA allows the decomposition of total genetic variance into hierarchical components corresponding to variation among populations, within populations, and within individuals (Excoffier et al., 1992; Roewer et al., 1996; Nievergelt et al., 2007). The statistical significance of variance components was assessed through permutation tests using genetic distance matrices derived from the SNP markers. The analysis was implemented in the R statistical environment using functions available in the dartR (Gruber et al., 2018) and ade4 packages (Dray and Dufour, 2007; Jombart, 2008).

## Results

### SNP dataset and filtering

Following quality filtering, the final DArTseq dataset comprised 91 individuals representing seven chicken populations and 43,017 high-quality SNP markers. Filtering was performed at the locus level; SNP markers with a locus call rate below 0.90 were excluded, whereas individual call rates were assessed descriptively and were not used as an exclusion criterion. The distribution of call rate and polymorphic information content (PIC) of the retained SNP markers is presented in Fig. 3. The average sequencing read depth was 10.04, and the overall proportion of missing genotypes was 4.08%. No monomorphic loci or loci containing exclusively missing data were detected, reflecting the preliminary quality filtering applied by the DArTsoft marker-calling pipeline prior to data delivery. Across all loci, genotype frequencies were 66.4% homozygous reference, 15.8% heterozygous, and 13.7% homozygous alternative, while 4.1% corresponded to missing genotypes. The mean call rate per individual was 0.959, ranging from 0.729 to 0.984, and the mean call rate per locus was also 0.959, indicating that 95.9% of all genotype calls were successfully assigned. The lowest individual call rate observed (0.729) indicated comparatively reduced genotyping completeness; however, this individual was retained because sample-level call rate was not used as an exclusion criterion.

**Fig. 3:**
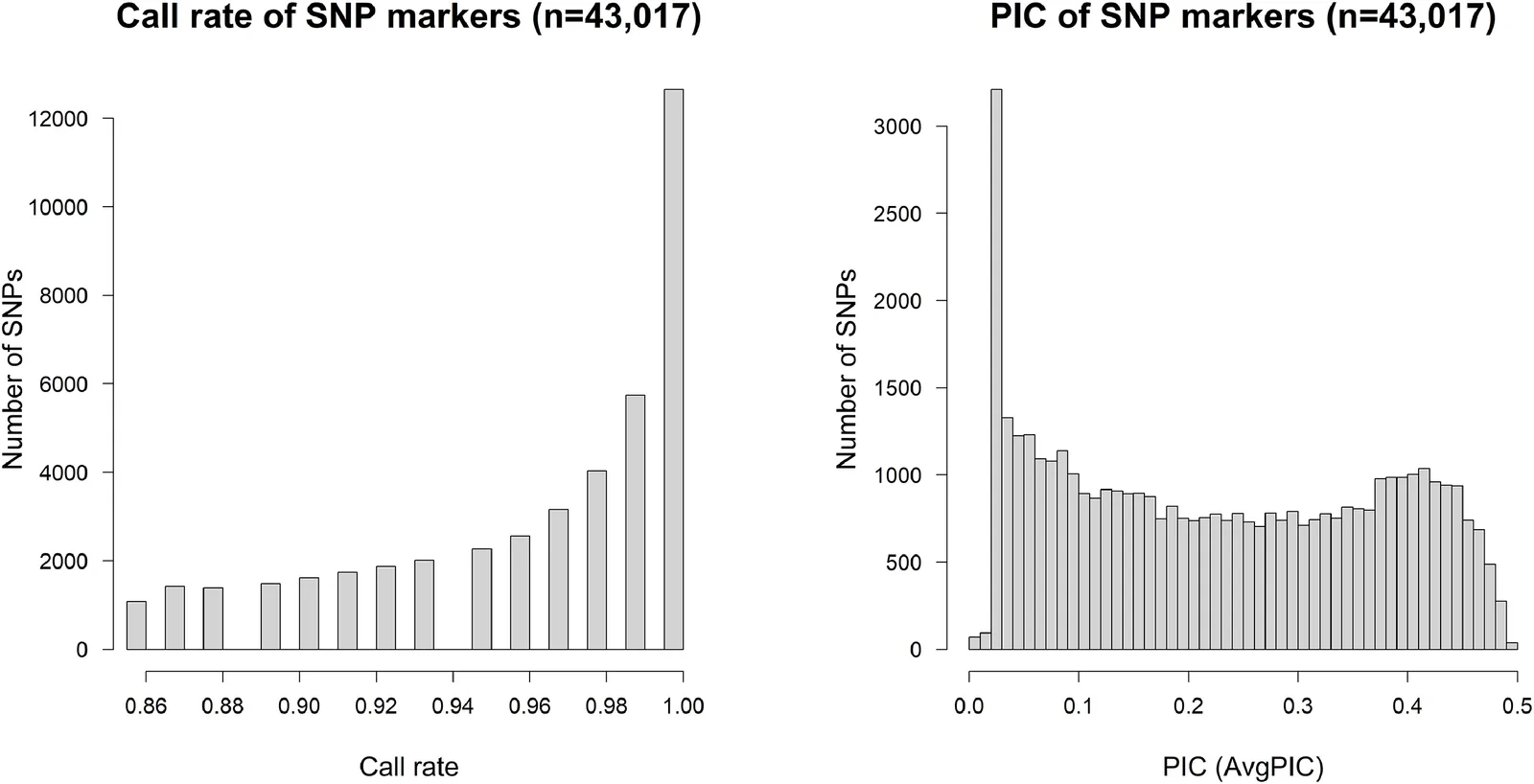
Distribution of call rate (a) and polymorphic information content (PIC) (b) of the 43,017 SNP markers retained after quality filtering of the DArTseq dataset. The histograms illustrate the quality and informativeness of the markers used for the downstream genetic diversity and population structure analyses.

### Genetic diversity within populations

Genetic diversity within populations was assessed using Ho, He, and the FIS. The estimates of these parameters for each population are presented in Table 2. Observed heterozygosity ranged from 0.130 to 0.186, with the lowest value recorded in Barred Plymouth Rock (Ho = 0.130) and the highest in Ketro (Ho = 0.186). Expected heterozygosity showed a wider range, varying from 0.166 in Barred Plymouth Rock to 0.236 in Ketro, indicating moderate levels of genetic diversity among the analyzed populations. The FIS values were generally positive in most populations, suggesting a relative deficit of heterozygotes. The highest FIS value was observed in Gallina de la Pasión (FIS = 0.336), followed by Kollonka (FIS = 0.263) and Kollonka de Aretes (FIS = 0.216). In contrast, ISA Brown presented a slightly negative FIS value (−0.049), indicating a small excess of heterozygotes relative to Hardy–Weinberg expectations. Overall, these results indicate intermediate genetic diversity across the studied populations, with variation in heterozygosity levels among breeds.

**Table 2:** Genetic diversity indices for the analyzed chicken populations based on 43,017 SNP markers.

Breed	N	Ho	He	FIS
Barred Plymouth Rock	13	0.130	0.166	0.220
Brahma	13	0.152	0.176	0.136
Gallina de la Pasión	13	0.147	0.222	0.336
ISA Brown	8	0.177	0.168	−0.049
Ketro	14	0.186	0.236	0.211
Kollonka	15	0.168	0.227	0.263
Kollonka de Aretes	15	0.180	0.230	0.216

### Distribution of SNP alleles

The distribution of alternate SNP allele counts across loci was evaluated for each population to assess patterns of allele frequency variation among breeds. The results are presented in Fig. 4. Across all populations, most loci showed low alternate allele counts, indicating that most SNP markers were present at relatively low frequencies within populations. This pattern is consistent with the presence of numerous loci with rare alleles across the genome. Similar distributions were observed among most populations, including Barred Plymouth Rock, Brahma, Gallina de la Pasión, ISA Brown, Ketro, Kollonka, and Kollonka de Aretes, where the highest frequencies were concentrated in the lowest allele count classes. However, slight differences in the distribution patterns among populations suggest variation in allele frequencies and levels of polymorphism. Overall, the allele count distributions indicate comparable levels of genomic variation across the analyzed populations.

**Fig. 4:**
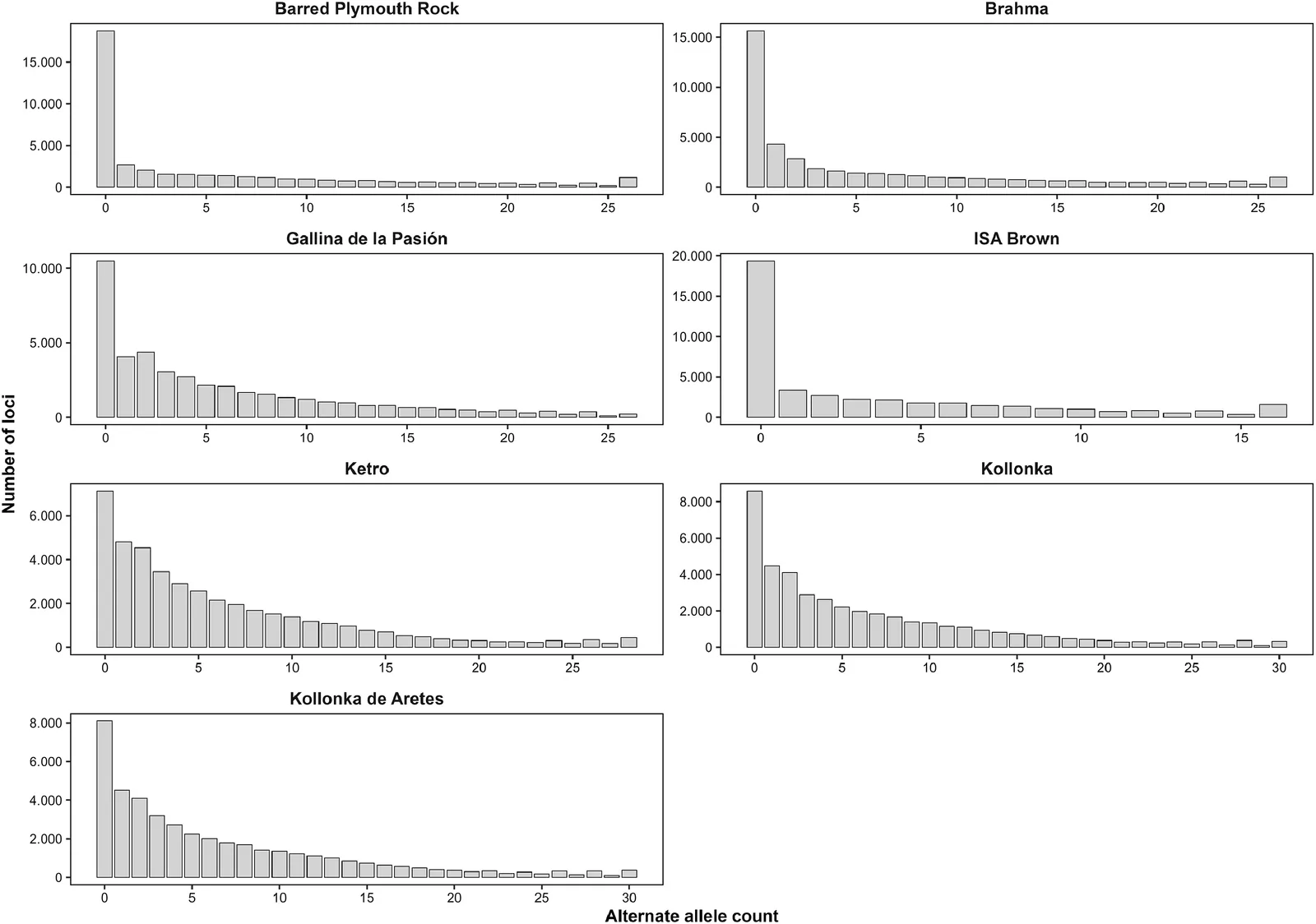
Distribution of alternate allele counts for the 43,017 SNP markers across the analyzed chicken populations. The histograms represent the number of loci for each alternate allele count class within each breed.

Multivariate analyses revealed clear genetic structuring among the analyzed populations. The PCA (Fig. 5) based on DArTseq SNPs showed that the first two components explained 14.37% of the total genetic variation (PC1 = 7.35%; PC2 = 7.02%). In the PC1–PC2 plane, a clear separation of Barred Plymouth Rock was observed, with individuals clustering toward negative values of PC1 and positive values of PC2, forming a well-defined cluster. Brahma was also differentiated from the remaining populations, being located toward positive values of both components. In contrast, the local populations Ketro, Kollonka, and Kollonka de Aretes clustered closely together and showed some overlap, suggesting a close genetic relationship among them. ISA Brown was positioned near this group, whereas Gallina de la Pasión tended to occupy negative values along PC2. These patterns were consistent with the results of the DAPC (Fig. 6), which identified seven population groups and retained six discriminant functions, achieving a high overall assignment success rate (90.11%). Classification accuracy was 100.00% for Barred Plymouth Rock, Brahma, Gallina de la Pasión, ISA Brown, and Kollonka de Aretes, whereas Ketro (78.57%) and Kollonka (60.00%) showed lower assignment accuracy. Overall, these results indicate a clear genetic differentiation between the commercial breeds and the local populations, as well as a closer genetic relationship among the Mapuche fowl varieties.

**Fig. 5:**
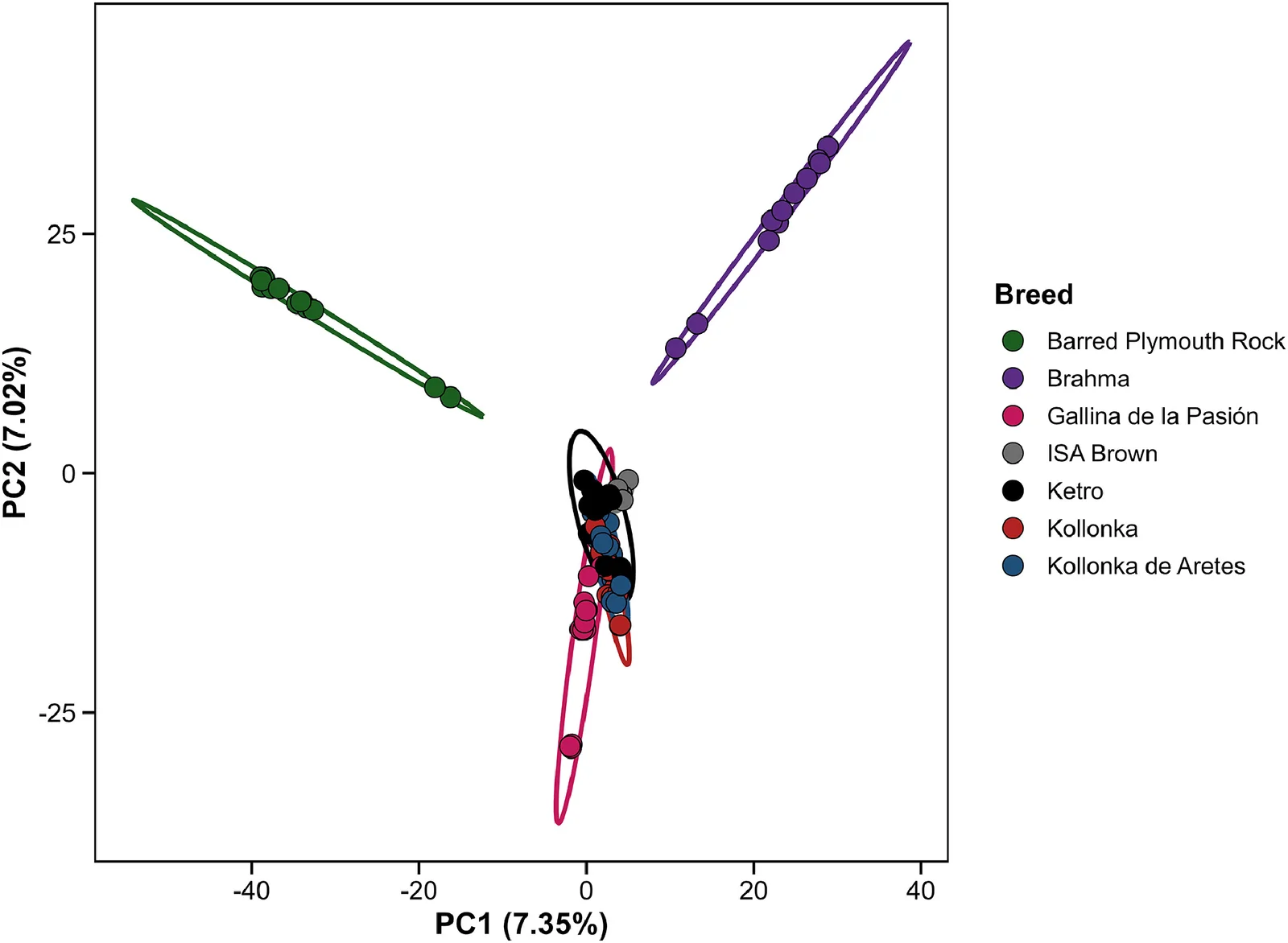
Principal Component Analysis (PCA) of the seven chicken populations based on DArTseq SNP markers.

**Fig. 6:**
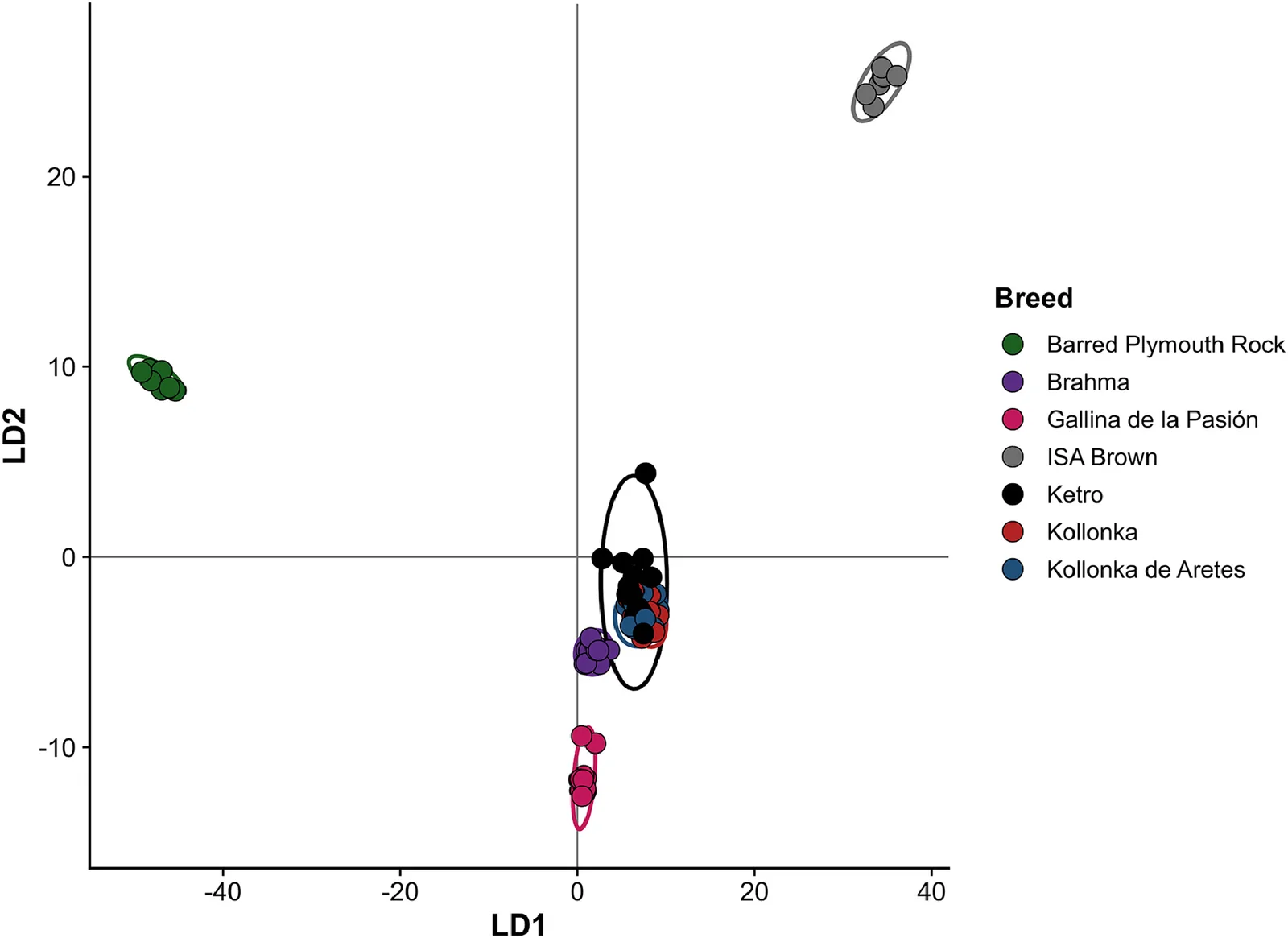
Discriminant Analysis of Principal Components (DAPC) showing genetic clustering of the seven chicken populations.

### Population structure and admixture

Population structure analysis based on sparse nonnegative matrix factorization revealed clear patterns of ancestry and admixture among the analyzed chicken populations. The evaluation of cross-entropy across values of K from 1 to 10 indicated variation in model fit among clustering solutions, with the lowest value observed at K = 7 (0.583840960020713), suggesting that this was the most informative number of ancestral populations for the dataset analyzed (Fig. 7; Table 3). The ancestry bar plots across K values showed that the commercial breeds tended to display more homogeneous ancestry profiles, whereas the Mapuche fowl populations shared similar ancestry components and exhibited closer genetic relationships (Fig. 8). Ketro, Kollonka, and Kollonka de Aretes showed broadly similar ancestry patterns across several K values, supporting their close genetic affinity. Gallina de la Pasión showed an intermediate pattern. Overall, the structure analyses were consistent with the PCA, DAPC, FST, and Nei’s genetic distance results, confirming clear differentiation between commercial breeds and the local Chilean populations, as well as a close relationship among the Mapuche fowl varieties.

**Fig. 7:**
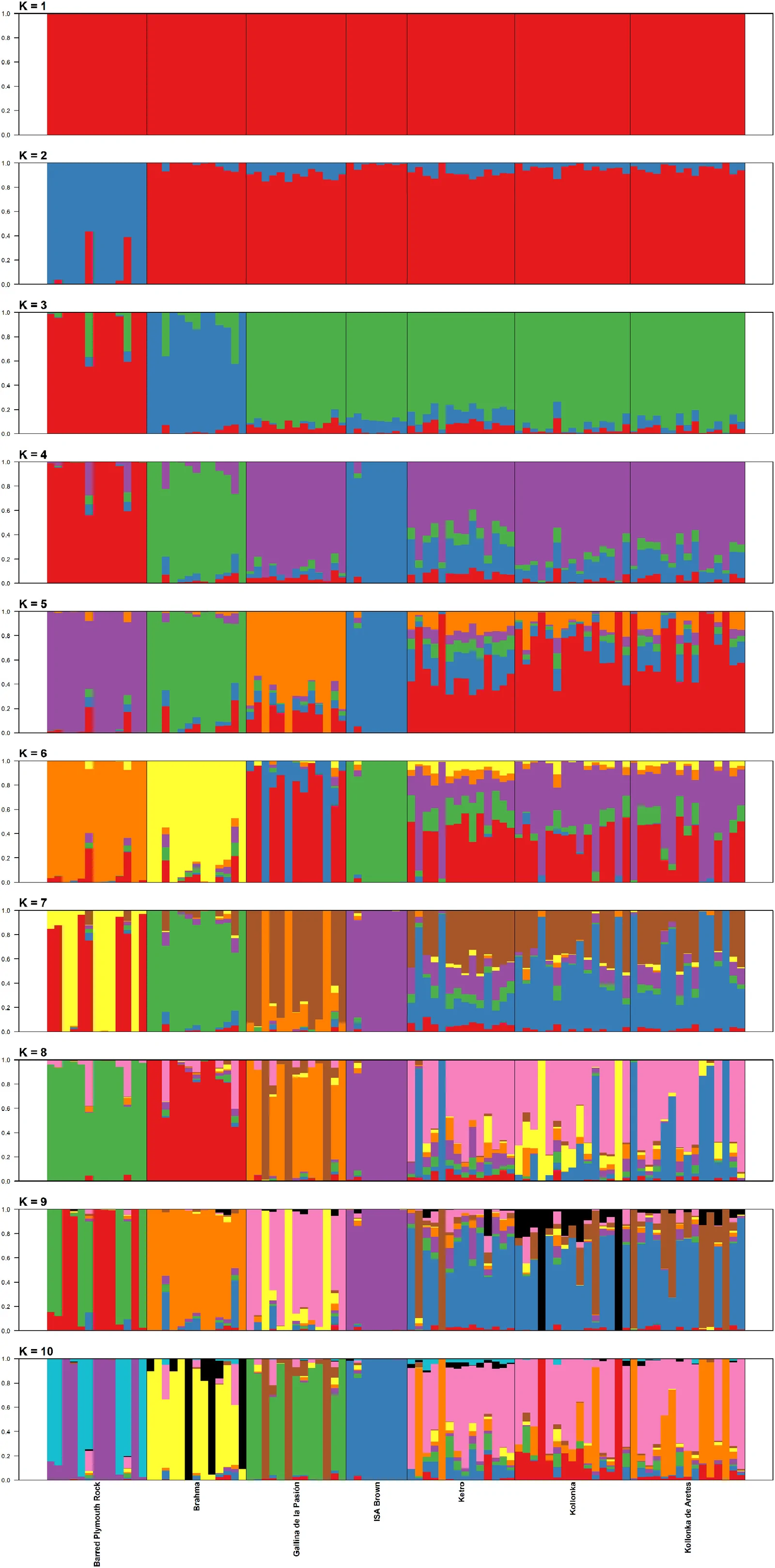
sNMF ancestry bar plots for K = 1 to 10 in the analyzed chicken populations.

**Table 3:** Cross-entropy values obtained for K = 1 to 10 in the sNMF analysis based on 43,017 DArTseq SNP markers.

K	Cross-Entropy
1	0.639143665558199
2	0.621297870857289
3	0.602258783519697
4	0.593601341836111
5	0.586610643611177
6	0.586257992229637
7	0.583840960020713
8	0.587145332669779
9	0.586493060946452
10	0.594489825649468

**Fig. 8:**
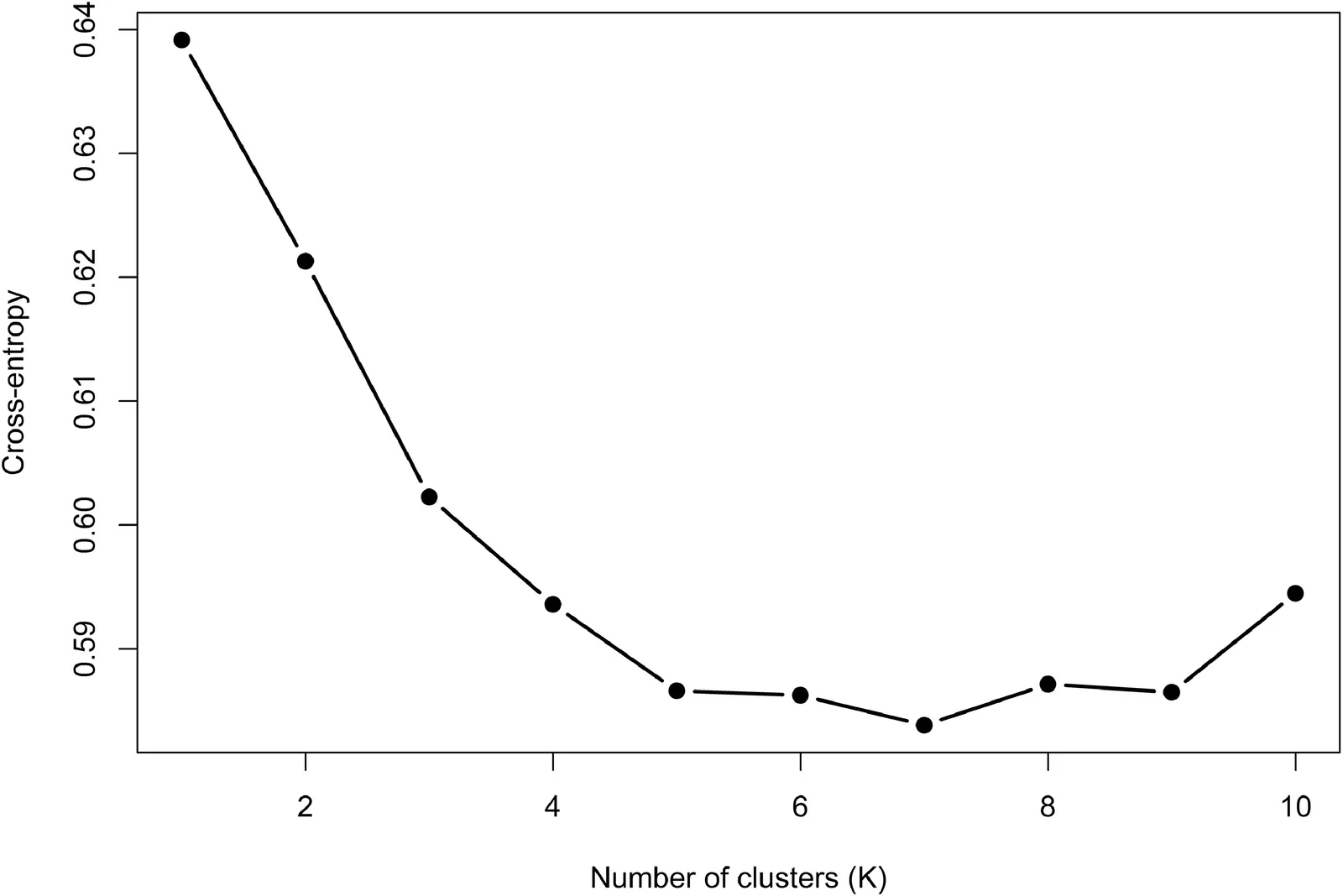
Cross-entropy values across K = 1 to 10 obtained in the sNMF analysis..

### Genetic differentiation among populations

Genetic differentiation among the analyzed populations was assessed using pairwise FST values based on DArTseq SNP markers (Table 4). Overall, moderate to high levels of genetic differentiation were observed among several populations. The highest differentiation was detected between ISA Brown and Barred Plymouth Rock (FST = 0.158), followed by Barred Plymouth Rock and Brahma (FST = 0.149), indicating substantial genetic divergence among these commercial breeds. In contrast, the lowest levels of differentiation were observed among the local populations Ketro, Kollonka, and Kollonka de Aretes, with FST values ranging from 0.026 to 0.032, suggesting a close genetic relationship among these populations. Intermediate levels of differentiation were detected between Gallina de la Pasión and the Mapuche fowl populations (FST ≈ 0.057–0.063). Overall, the results indicate a clear genetic differentiation between the commercial breeds and the local Chilean populations, while the Mapuche fowl varieties exhibit relatively low genetic divergence, consistent with their shared genetic background.

**Table 4:** Pairwise FST values among chicken populations based on DArTseq SNP markers.

Population	Barred Plymouth Rock	Brahma	Gallina de la Pasión	ISA Brown	Ketro	Kollonka	Kollonka de Aretes
Barred Plymouth Rock	0	–	–	–	–	–	–
Brahma	0.1487	0	–	–	–	–	–
Gallina de la Pasión	0.1224	0.1195	0	–	–	–	–
ISA Brown	0.1582	0.1422	0.1219	0	–	–	–
Ketro	0.0884	0.0805	0.0571	0.0765	0	–	–
Kollonka	0.1019	0.0939	0.0628	0.0930	0.0316	0	–
Kollonka de Aretes	0.0967	0.0890	0.0618	0.0868	0.0265	0.0286	0

### Analysis of molecular variance (AMOVA)

The AMOVA revealed that most of the genetic variation was distributed within populations, accounting for 72.93% of the total variance, whereas 13.25% was attributed to differences among populations and 13.82% to differences within individuals (Table 5). The latter component reflects the diploid nature of the SNP markers analyzed. These results indicate that, although a considerable proportion of genetic diversity is shared among populations, a detectable level of genetic structuring exists among the studied chicken populations. This pattern is consistent with the differentiation observed in PCA, DAPC, and FST analyses.

**Table 5:** Analysis of Molecular Variance (AMOVA) based on DArTseq SNP markers.

Source of variation	Percentage of variation
Among populations	13.25%
Within populations	72.93%
Within individuals	13.82%
Total	100.00

### Phylogenetic relationships

Phylogenetic relationships among the analyzed chicken populations were inferred using Nei’s genetic distance (1972) based on DArTseq SNP markers. Pairwise genetic distances are presented in Table 6, and the resulting clustering pattern is illustrated in the Neighbor-Joining tree (Fig. 9). The smallest genetic distances were observed among the Mapuche fowl populations, particularly between Ketro and Kollonka de Aretes (0.0177) and between Kollonka and Kollonka de Aretes (0.0190), indicating a very close genetic relationship among these populations. Similarly, Ketro and Kollonka also showed a low genetic distance (0.0217), further supporting their shared genetic background. Intermediate genetic distances were observed between Gallina de la Pasión and the Mapuche fowl populations (approximately 0.044–0.048), suggesting a moderate level of genetic differentiation. In contrast, larger genetic distances were detected between the commercial breeds and the local populations. The highest genetic distance was observed between Barred Plymouth Rock and Brahma (0.1084), followed by Barred Plymouth Rock and ISA Brown (0.1070). The Neighbor-Joining tree clearly reflects these relationships, grouping the Mapuche fowl populations (Ketro, Kollonka, and Kollonka de Aretes) within a closely related cluster, while the commercial breeds form more distant branches. Overall, these results confirm the genetic differentiation between commercial chicken breeds and the local Chilean populations, while also highlighting the close genetic relationship among the Mapuche fowl varieties.

**Table 6:** Pairwise Nei genetic distances among chicken populations based on DArTseq SNP markers.

Population	Barred Plymouth Rock	Brahma	Gallina de la Pasión	ISA Brown	Ketro	Kollonka	Kollonka de Aretes
Barred Plymouth Rock	0	–	–	–	–	–	–
Brahma	0.1084	0	–	–	–	–	–
Gallina de la Pasión	0.0957	0.0983	0	–	–	–	–
ISA Brown	0.1070	0.0997	0.0940	0	–	–	–
Ketro	0.0656	0.0615	0.0442	0.0532	0	–	–
Kollonka	0.0771	0.0734	0.0484	0.0673	0.0217	0	–
Kollonka de Aretes	0.0728	0.0690	0.0481	0.0619	0.0177	0.0190	0

**Fig. 9:**
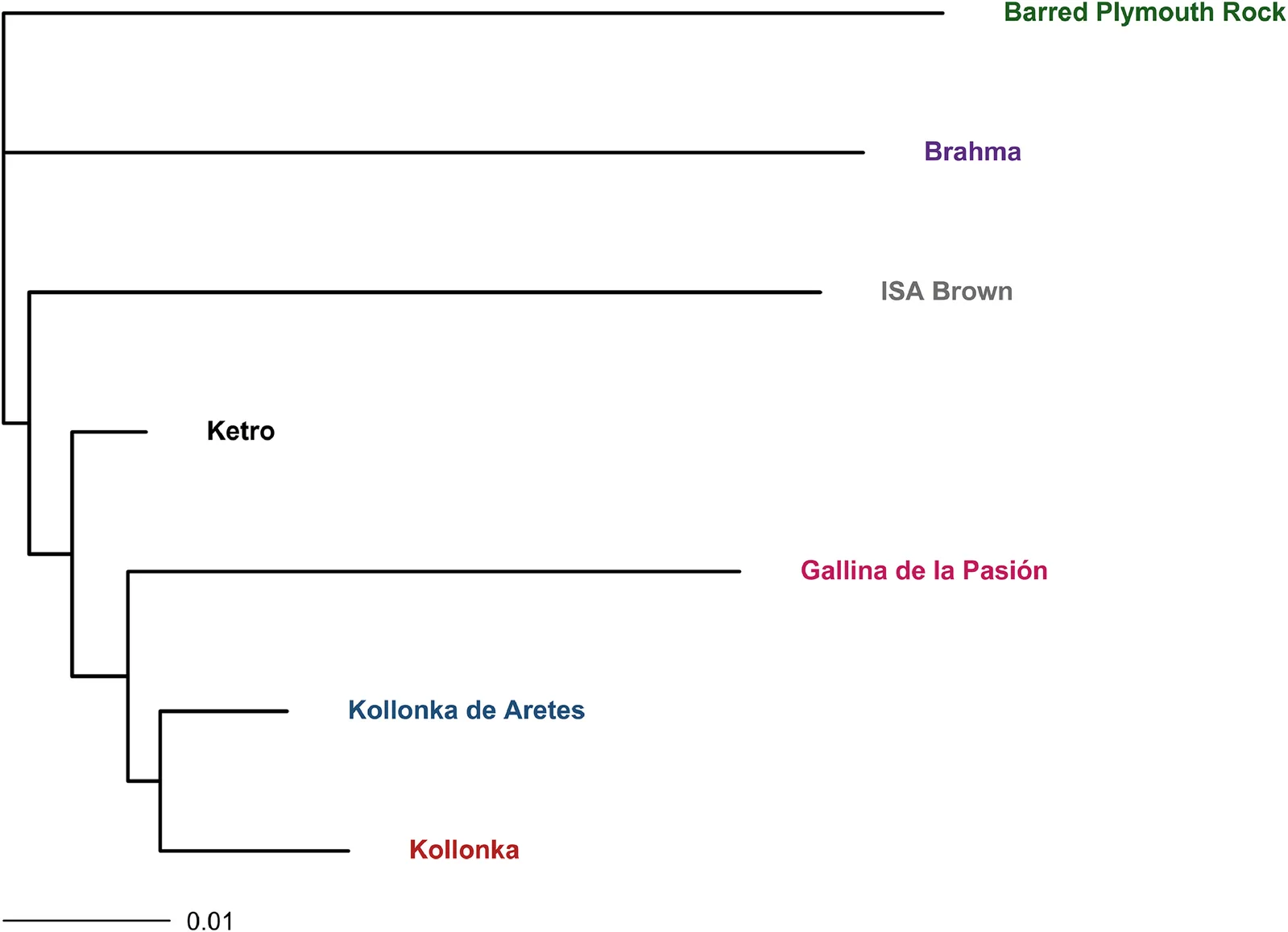
Neighbor-Joining phylogenetic tree based on Nei genetic distances among the analyzed chicken populations using DArTseq SNP markers.

## Discussion

The present study assessed the genetic diversity and population structure of seven chicken populations using 43,017 SNPs generated through DArTseq technology, providing substantial genomic resolution for examining patterns of genetic variation. The use of tens of thousands of genome-wide SNPs enables a more accurate characterization of population differentiation than approaches based on microsatellites or reduced marker panels (Cendron et al., 2021; Ajmone-Marsan et al., 2023; Yang et al., 2025). Previous genomic studies in domestic chickens have shown that datasets comprising 20,000 to 60,000 SNPs provide sufficient resolution to characterize genetic diversity, population structure, and phylogenetic relationships among both commercial breeds and local populations (Zhang et al., 2018; Eusebi et al., 2019).

The analyzed populations exhibited moderate levels of genetic diversity. Observed heterozygosity ranged from 0.130 to 0.186, whereas expected heterozygosity ranged from 0.166 to 0.236. The highest levels of genetic diversity were found in Ketro (He = 0.236), Kollonka de Aretes (He = 0.230), and Kollonka (He = 0.227), while the lowest values were recorded in Barred Plymouth Rock (He = 0.166) and Brahma (He = 0.176). These findings indicate that the local populations, particularly the Mapuche fowl varieties, retain greater genetic diversity than some of the commercial breeds included in this study. Similar patterns have been reported for local chicken populations worldwide, as populations maintained under traditional and extensive production systems often preserve greater genetic variability because they have not been subjected to intensive artificial selection (Adomako et al., 2024; Mujyambere et al., 2024, 2025; Mogano et al., 2025).

Observed heterozygosity was lower than expected heterozygosity in most populations, resulting in positive FIS values. This pattern indicates a relative deficit of heterozygotes, which may reflect population substructure, non-random mating, or mating among related individuals. The highest FIS value was observed in Gallina de la Pasión (FIS = 0.336), followed by Kollonka (FIS = 0.263) and Kollonka de Aretes (FIS = 0.216). A similar heterozygote deficit has been reported in indigenous African chicken populations, in which observed heterozygosity ranged from 0.316 to 0.339, expected heterozygosity ranged from 0.388 to 0.405, and FIS values ranged from 0.173 to 0.181 (Mujyambere et al., 2025). By contrast, ISA Brown exhibited a negative FIS value (−0.049), indicating a slight excess of heterozygotes. Similar results have been described in highly managed commercial lines, in which crosses between genetically differentiated parental lines can produce greater heterozygosity than that observed in local populations (Cendron et al., 2020).

The genetic differentiation analyses revealed moderate to high divergence among some populations. The highest FST values were observed between ISA Brown and Barred Plymouth Rock (FST = 0.158) and between Barred Plymouth Rock and Brahma (FST = 0.149), indicating substantial differentiation among these commercial breeds. In contrast, Ketro, Kollonka, and Kollonka de Aretes showed very low levels of differentiation (FST = 0.026–0.032), demonstrating a close genetic relationship among the Mapuche fowl varieties. Comparable levels of differentiation have been reported among five Chinese indigenous chicken breeds, with pairwise FST values ranging from 0.03 to 0.10, whereas comparisons involving European and North American breeds reached values of up to 0.19 (Nie et al., 2019). Greater differentiation was reported among eight Italian local chicken breeds, for which pairwise FST values ranged from 0.114 to 0.565; however, the two Polverara varieties showed the lowest differentiation (FST = 0.114), consistent with their shared breed origin (Cendron et al., 2021). Similarly, genome-wide comparisons among Chinese local and commercial chicken populations yielded pairwise FST values ranging from 0.015 to 0.145, with the lowest differentiation observed between Jining Bairi and Langya chickens (FST = 0.015) and the highest between Wenshang Barred and Shouguang chickens (FST = 0.145) (Jie et al., 2023). According to Wright (1951), FST values below 0.05 indicate low genetic differentiation, whereas values from 0.05 to 0.15 represent moderate differentiation. Accordingly, the low values observed among the Mapuche fowl varieties suggest that they share a common genetic background and have likely experienced gene flow over time (Díaz-Matus de la Parra et al., 2024).

The multivariate analyses also revealed a distinct pattern of genetic structure among the populations. PCA clearly separated the commercial breeds from the local populations, whereas Ketro, Kollonka, and Kollonka de Aretes clustered closely, reflecting their genetic proximity (Díaz-Matus de la Parra et al., 2024). Comparable patterns have been reported in genomic studies of domestic chickens, in which local populations tend to cluster according to their geographic origin or shared genetic history. In contrast, commercial breeds usually form distinct clusters because of intensive artificial selection and controlled breeding practices (Cendron et al., 2020, 2021; Perini et al., 2020). Consistent with the PCA results, DAPC produced a more pronounced separation among the predefined groups and confirmed that the Mapuche subtypes form a genetically cohesive group that is differentiated from the commercial breeds analyzed.

The sNMF analysis provided further support for the genetic relationships identified among the populations. Cross-entropy reached its minimum at K = 7 (0.583840960020713), indicating that seven ancestral components provided the best-supported representation of the genetic structure in this dataset. Across K = 1–10, commercial breeds generally exhibited more homogeneous ancestry profiles, whereas the Mapuche fowl varieties shared multiple ancestry components. Ketro, Kollonka, and Kollonka de Aretes displayed broadly similar ancestry patterns across several K values, further supporting their close genetic affinity. These genomic findings are consistent with the low pairwise FST values, short Nei’s genetic distances, and overlapping distribution of these populations in the PCA and DAPC. They also extend the results of the only previous study specifically addressing the genetic structure of Mapuche fowl, which used microsatellite markers and similarly identified substantial shared ancestry and limited differentiation among Mapuche populations (Díaz-Matus de la Parra et al., 2024). Comparable patterns have been reported in Italian local chickens. Perini et al. (2020) found that some of the 13 breeds examined shared genetic components, reflecting common origins, historical gene flow, and incomplete reproductive isolation. Likewise, a genome-wide analysis of eight Italian local breeds showed that closely related varieties, particularly Polverara Bianca and Polverara Nera, shared ancestry components and exhibited limited differentiation, whereas more distinct breeds formed comparatively homogeneous genetic clusters (Cendron et al., 2021). These findings illustrate that phenotypically recognized local varieties do not necessarily represent fully independent genetic populations, particularly when they share a common origin or have experienced historical crossbreeding.

Gallina de la Pasión exhibited an intermediate ancestry profile, suggesting both differentiation from the Mapuche fowl varieties and partial sharing of ancestry components. Because this is the first genome-wide population structure study to include Gallina de la Pasión, its ancestry pattern cannot be directly compared with previous genomic evidence from this population. Nevertheless, complex ancestry and admixture have also been reported in other dwarf and bantam populations. For example, genomic analyses of traditional Dutch chickens revealed a complex and admixed population structure shaped by selection, geographic history, and crossbreeding, including shared ancestry between standard breeds and their bantam counterparts (Wu et al., 2021, 2023). Therefore, the intermediate position of Gallina de la Pasión should be interpreted cautiously as evidence of a distinct but admixed genomic background rather than as proof of a particular origin. Overall, the sNMF results indicate that the Mapuche fowl varieties are not genetically isolated populations but closely related components of a broader population complex that retains substantial shared ancestry.

The AMOVA showed that 72.93% of the total genetic variation occurred within populations, whereas 13.25% was attributable to differences among populations. This distribution is consistent with previous population genetic studies in chickens, in which most genetic variability is generally found within rather than among populations (Pham et al., 2013; Strillacci et al., 2017; de Carvalho et al., 2020; Mujyambere et al., 2025). The smaller proportion of variation attributed to differences among populations indicates that, despite the differentiation detected between the commercial breeds and local populations, these populations retain a substantial shared genetic background.

Phylogenetic relationships inferred from Nei’s genetic distances (1972) were consistent with the patterns identified by the differentiation, multivariate, and ancestry analyses. The shortest distances were observed among the Mapuche fowl varieties, particularly between Ketro and Kollonka de Aretes (0.0177), Kollonka and Kollonka de Aretes (0.0190), and Ketro and Kollonka (0.0217). Accordingly, these populations clustered within the same clade in the Neighbor-Joining tree, supporting their close genetic affinity and probable derivation from a shared genetic background. This result extends previous microsatellite-based evidence indicating limited genetic differentiation and substantial genetic similarity among Mapuche fowl populations (Díaz-Matus de la Parra et al., 2024). Comparable phylogenetic patterns have been reported for other local chicken populations. Among Italian breeds, the two Polverara varieties, Bianca and Nera, showed limited differentiation and clustered closely, consistent with their common origin, whereas more differentiated breeds occupied separate positions in the genetic analyses (Cendron et al., 2021). Similarly, genome-wide SNP analyses of Chinese chickens showed that indigenous breeds with greater genetic affinity tended to cluster together, whereas European and North American commercial breeds formed more distant groups, reflecting differences in their demographic and selection histories (Nie et al., 2019). Jie et al. (2023) also identified close relationships among some Chinese local populations, including Jining Bairi and Langya chickens, which exhibited the lowest pairwise differentiation in their dataset and showed consistent proximity in the population-level analyses. In the present study, the greatest Nei’s genetic distances were observed between Barred Plymouth Rock and Brahma (0.1084) and between Barred Plymouth Rock and ISA Brown (0.1070), and these populations were located on more distant branches of the tree. This separation likely reflects their different breeding histories and the effects of artificial selection rather than simply their classification as commercial populations (Nie et al., 2019). Overall, the concordance among the Neighbor-Joining tree, Nei’s genetic distances, FST, PCA, DAPC, and sNMF results provides robust evidence that Ketro, Kollonka, and Kollonka de Aretes constitute a closely related population complex that is genetically distinguishable from the commercial breeds and line included in this study.

From this perspective, the term fowl, as applied to Mapuche chickens, should not be interpreted as denoting a formally standardized breed in the conventional zootechnical sense. Rather, the Mapuche fowl appears to represent a heterogeneous population complex derived from a shared ancestral gene pool. In contrast to standardized breeds, whose phenotypic and genetic uniformity has generally been reinforced through controlled mating, reproductive isolation, and sustained directional selection, Mapuche fowl populations have been shaped primarily by traditional husbandry, relatively limited directed selection, and historical gene flow among their principal subtypes, including Kollonka, Kollonka de Aretes, and Ketro (Díaz-Matus de la Parra et al., 2024). The low pairwise genetic differentiation, close clustering in the PCA and DAPC, and extensive sharing of ancestry components detected in the present study indicate that these subtypes are not fully independent genetic entities. Instead, they constitute closely related populations with a substantial shared genetic background. Similar patterns have been documented in genomic surveys of locally managed chickens, in which historically recognized populations may retain distinctive phenotypic identities despite incomplete genetic separation resulting from common ancestry and ongoing gene flow (Malomane et al., 2019; Cendron et al., 2021; Mujyambere et al., 2024, 2025). Therefore, the present results support the interpretation of Mapuche fowl as a genetically coherent population complex rather than as a collection of completely differentiated breeds.

Unlike the Mapuche fowl subtypes, Gallina de la Pasión—locally known as “Kike” or “Kikiriki”—displayed a more differentiated genetic profile in the analyses conducted here. This population corresponds to a dwarf chicken traditionally classified within the bantam category and is primarily maintained for ornamental purposes. Gallina de la Pasión has been recognized as one of the historical chicken populations present in Chile, and mitochondrial evidence has suggested that some Chilean chickens carry maternal lineages related to Asian populations (Gongora et al., 2008). Nevertheless, the available evidence does not support considering Gallina de la Pasión a genetically isolated or formally standardized breed. Its sharing of mitochondrial haplotypes with Mapuche and other Chilean local chickens is compatible with either common maternal ancestry or historical introgression among backyard populations. In the present study, Gallina de la Pasión exhibited an intermediate ancestry profile and was differentiated from Ketro, Kollonka, and Kollonka de Aretes in the multivariate and population structure analyses. This pattern suggests that it has retained a recognizable genetic and phenotypic identity while also showing evidence of shared ancestry and admixture. Comparable results have been reported in other indigenous and bantam chicken populations, where phenotypically recognizable groups were genetically distinguishable but still displayed varying degrees of admixture with neighboring or introduced populations (Bortoluzzi et al., 2018; Malomane et al., 2019; Wu et al., 2021, 2023; Demir et al., 2025). Gallina de la Pasión should therefore be interpreted cautiously as a relatively differentiated local population within Chilean backyard production systems, rather than as a completely isolated genetic group.

Beyond characterizing genetic diversity and population structure, these findings have direct implications for population assignment, reproductive management, and conservation planning. The close relationships among Ketro, Kollonka, and Kollonka de Aretes, together with their low genetic differentiation and extensive sharing of ancestry components, support their management as components of a single, coherent genetic complex (Díaz-Matus de la Parra et al., 2024). However, recognizing their shared genomic background does not necessarily preclude the conservation of their distinctive phenotypic and cultural attributes. Accordingly, conservation programs should preserve both the overall genetic diversity of Mapuche fowl and the characteristic traits associated with each subtype. Genome-wide SNP datasets provide substantially greater resolution than small microsatellite panels for quantifying genetic differentiation, detecting admixture, assigning individuals to populations, and identifying animals suitable for conservation or breeding programs (Malomane et al., 2019; Adomako et al., 2023, 2024; Jie et al., 2023; Mujyambere et al., 2024, 2025; Demir et al., 2025). Such applications are particularly relevant in backyard systems, where uncontrolled mating, small flock sizes, and the introduction of commercial or exotic chickens may alter allele frequencies and progressively erode local genetic identity (Cendron et al., 2020; Mujyambere et al., 2025). Therefore, genomic information should be incorporated into reproductive management through the selection of genetically representative and minimally related breeders, the monitoring of introgression, and the establishment of population-specific conservation objectives.

From a conservation perspective, the relatively high genetic diversity retained by Mapuche fowl reinforces its value as an animal genetic resource and supports FAO priorities for the characterization and preservation of local populations (FAO, 2011, 2013; Thieme et al., 2014). Genomic tools could assist in selecting representative and minimally related individuals, establishing conservation nuclei, and monitoring inbreeding. Future studies incorporating genomic inbreeding coefficients and runs of homozygosity (ROH) would provide further insight into demographic history and accumulated inbreeding, thereby supporting evidence-based management decisions (Macharia et al., 2024).

Developing a reduced diagnostic SNP panel for population assignment would facilitate the practical application of these genomic findings (Seo et al., 2021; Zhang et al., 2025). Such a panel could provide rapid and cost-effective identification of Mapuche fowl, supporting traceability, certification, and potential denomination-of-origin initiatives associated with distinctive products such as blue-shelled eggs. Overall, these findings provide a scientific basis for genomic management, population certification, and the sustainable conservation of this valuable local genetic resource.

## Conclusions

Using 43,017 DArTseq SNPs, we provide the first genome-wide characterization of the Chilean Mapuche fowl. The three Mapuche varieties (Ketro, Kollonka, Kollonka de Aretes) retained the highest genetic diversity and formed a single, closely related genetic group (FST = 0.026–0.032), clearly differentiated from commercial breeds. These results confirm the Mapuche fowl as a distinctive local genetic resource with a shared ancestral background, and provide a genomic basis for its conservation, breed assignment, and sustainable management.

## Author Contributions

**Mario Díaz-Matus de la Parra:** Contributed substantially to the conceptualization and design of the study, methodology, investigation, formal analysis, data curation, validation, software, visualization, and writing of the original draft, as well as critical revision of the manuscript. He approves the final version of the manuscript and agrees to be accountable for all aspects of the work. **Karla Inostroza:** Contributed substantially to the methodology and investigation, as well as to the writing of the original draft and critical revision of the manuscript. She approves the final version of the manuscript and agrees to be accountable for all aspects of the work. **José Antonio Alcalde:** Contributed substantially to the conceptualization and design of the study, methodology, and investigation, as well as to the writing of the original draft and critical revision of the manuscript. He approves the final version of the manuscript and agrees to be accountable for all aspects of the work. **Daniela Bustos-Korts:** Contributed to the formal analysis and critically reviewed the manuscript for important intellectual content. She approves the final version of the manuscript and agrees to be accountable for all aspects of the work. **Giovanni Larama:** Contributed substantially to the methodology, formal analysis, data curation, validation, visualization, and writing of the original draft. He approves the final version of the manuscript and agrees to be accountable for all aspects of the work. **Silvana Bravo:** Contributed substantially to the conceptualization and design of the study, methodology, investigation, validation, supervision, resources, project administration, and funding acquisition, as well as to the writing of the original draft and critical revision of the manuscript. She approves the final version of the manuscript and agrees to be accountable for all aspects of the work.

## Ethics declaration

This study was conducted in accordance with the following guidelines for animal welfare and/or reporting: Institutional Committee for Animal Care and Use (CICUA) of the Austral University of Chile. This study was approved by the Institutional Committee for Animal Care and Use (CICUA) of the Austral University of Chile. (Approval No. N°570/2025 of January 8, 2025).

## Disclosures

The authors declare that they have no known competing financial interests or personal relationships that could have appeared to influence the work reported in this study.
